# Enhancing the diversity of breeding invertebrates within field margins of intensively managed grassland: Effects of alternative management practices

**DOI:** 10.1002/ece3.3302

**Published:** 2017-10-19

**Authors:** Rochelle A. Fritch, Helen Sheridan, John A. Finn, Stephen McCormack, Daire Ó hUallacháin

**Affiliations:** ^1^ UCD School of Agriculture & Food Science University College Dublin Dublin Ireland; ^2^ Teagasc Environment Research Centre Wexford Ireland

**Keywords:** agri‐environment schemes, biodiversity, conservation, grassland management, grazing, habitat heterogeneity, natural regeneration, parasitoid, wildflower seed mixture

## Abstract

Severe declines in biodiversity have been well documented for many taxonomic groups due to intensification of agricultural practices. Establishment and appropriate management of arable field margins can improve the diversity and abundance of invertebrate groups; however, there is much less research on field margins within grassland systems. Three grassland field margin treatments (fencing off the existing vegetation “fenced”; fencing with rotavation and natural regeneration “rotavated” and; fencing with rotavation and seeding “seeded”) were compared to a grazed control in the adjacent intensively managed pasture. Invertebrates were sampled using emergence traps to investigate species breeding and overwintering within the margins. Using a manipulation experiment, we tested whether the removal of grazing pressure and nutrient inputs would increase the abundance and richness of breeding invertebrates within grassland field margins. We also tested whether field margin establishment treatments, with their different vegetation communities, would change the abundance and richness of breeding invertebrates in the field margins. Exclusion of grazing and nutrient inputs led to increased abundance and richness in nearly all invertebrate groups that we sampled. However, there were more complex effects of field margin establishment treatment on the abundance and richness of invertebrate taxa. Each of the three establishment treatments supported a distinct invertebrate community. The removal of grazing from grassland field margins provided a greater range of overwintering/breeding habitat for invertebrates. We demonstrate the capacity of field margin establishment to increase the abundance and richness in nearly all invertebrate groups in study plots that were located on previously more depauperate areas of intensively managed grassland. These results from grassland field margins provide evidence to support practical actions that can inform Greening (Pillar 1) and agri‐environment measures (Pillar 2) of the Common Agricultural Policy (CAP). Before implementing specific management regimes, the conservation aims of agri‐environment measures should be clarified by defining the target species or taxonomic groups.

## 1 INTRODUCTION

Severe declines in biodiversity have been well documented for many groups due to intensification of agricultural practices (Robinson & Sutherland, 2002; Tscharntke, Klein, Kruess, Steffan‐Dewenter, & Thies, 2005). These declines have occurred across all trophic levels, including plants, invertebrates, birds and mammals (Benton, Bryant, Cole, & Crick, 2002; Conrad, Warren, Fox, Parsons, & Woiwod, 2006; Donald, Sanderson, Burfield, & van Bommel, 2006). Increased homogenization of the farmed landscape, due to intensification and specialization of agricultural management practices, has been cited as the primary cause of losses in diversity (Tscharntke et al., 2005). Many conservation efforts in agricultural landscapes aim to improve habitat heterogeneity.

Manipulation of field margins in arable farm systems can improve the diversity and abundance of different invertebrate groups by increasing habitat heterogeneity (Haaland, Naisbit, & Bersier, 2011). This practice has been employed in Europe through agri‐environment schemes with the aim of mitigating the negative impacts of intensive agriculture (Meek et al., 2002). However, there is less research available on field margins within grassland systems (but see Haysom, McCracken, Foster, & Sotherton, 2004; Cole, McCracken, Baker, & Parish, 2007; Woodcock et al., 2007; Sheridan, Finn, Culleton, & O'Donovan, 2008; Potts et al., 2009; Fritch, Sheridan, Finn, Kirwan, & Ó hUallacháin, 2011). While pastoral systems are perceived to be less ecologically detrimental than arable systems; extensive loss of diversity has also been documented from these (Donald et al., 2006; Wilson, Morris, Arroyo, Clark, & Bradbury, 1999). Changes in grassland management including increased fertiliser use and stocking rates, homogenization of the sward, and a move from hay to silage, have led to losses of invertebrate diversity (Atkinson et al., 2005; Kruess & Tscharntke, 2002; Purvis & Curry, 1981). Thus, pastures and their margins are an important agricultural habitat to target within agri‐environment schemes.

There is also a lack of research investigating the invertebrates that actually breed in field margins (however, see Thomas & Marshall, 1999). Even fewer studies use trapping methods that eliminate potentially highly mobile species from their data. For example, pitfall trapping is commonly used to collect invertebrates in agricultural invertebrate surveys despite many ground‐dwelling species being highly mobile; furthermore, there may be sampling biases associated with differing sward densities (Melbourne, 1999). Studies on arable field margin invertebrates have primarily focused on very mobile insects, e.g., bumblebees, ground beetles and butterflies. These groups benefit from the establishment of wildflower margins in arable land (Haaland et al., 2011). However, there is debate as to whether field margins act as an “ecological trap,” a “low‐quality habitat that organisms prefer over superior habitats,” reducing overall species fitness and reproduction (Battin, 2004).

In this study, emergence traps were used to monitor resident field margin invertebrate species from spring through the summer season. Emergence traps provide one of the most quantitative forms of invertebrate sampling. This method ensures that the specimens are collected from the experimental unit of interest and are not migrant species. Emergence traps sample invertebrates living in and emerging from the vegetation/soil underneath the trap. They are an effective method to catch a range of less mobile invertebrates groups, such as Diptera, Araneae, Hemiptera, and parasitic Hymenoptera. Breeding success was measured by the emergence of invertebrates from the field margin sward/soil and can be considered a measure of habitat quality for invertebrate populations. Emergence traps also facilitate the calculation of invertebrates found per unit area (e.g., m^−2^). There is less bias associated with emergence trapping compared to either pitfall or suction sampling, when sampling occurs at varying sward densities (Sunderland et al., 1995). Despite these benefits, this method is relatively rarely used in terrestrial invertebrate sampling (but see Hanson, Palmu, Birkhofer, Smith, & Hedlund, 2016; Sheridan et al., 2008; Büchs, Harenberg, Zimmermann, & Weiss, 2003).

We tested two hypotheses regarding invertebrates breeding in grassland field margins using a manipulation experiment. Firstly, we tested whether a removal of grazing pressure would increase the abundance and richness of breeding invertebrates in the field margins. Second, we tested whether field margin establishment treatments, with differing levels of plant species richness, would increase the abundance, richness and community of breeding invertebrates in grassland field margins.

## 2 MATERIALS AND METHODS

### 2.1 Site location and description

The experiment was conducted on a lowland dairy farm on clay‐loam soil at the Teagasc Research Centre, Johnstown Castle, Co. Wexford, Ireland. All internal farm hedgerows were removed in the 1970s and paddocks separated by electric fencing. The area was sown with a mid‐season yielding variety of *Lolium perenne* approximately 4 years before the experiment was established in 2002. Paddocks were grazed at a stocking rate of between 2.4–2.8 livestock units ha^−1^ by a Friesian dairy herd on a 21‐day rotation, and cut for silage in alternate years. Between 200–375 kg ha^−1^ Nitrogen (N), 0–50 kg ha^−1^ Phosphorous (P) and 0–75 kg ha^−1^ Potassium (K) were applied annually to the swards adjacent to the experimental plots from 2002–2008. Further details are supplied in Sheridan et al. (2008) and Fritch et al. (2011).

### 2.2 Experimental design

This experiment was part of a larger study that investigated the impact of field margin establishment methods and management on farmland biodiversity. Here, the larger experiment is introduced, then a description of plots sampled in this invertebrate experiment is outlined. A stratified, randomised replicated split–split plot field margin experiment was established in 2002 (see Sheridan et al., 2008) comparing field margin width, establishment method, and management method. There were three replicates of all treatments (see diagram of field experiment in Fig S1). The experimental main plot was the width treatment (1.5, 2.5, and 3.5 m), and this was split into three establishment treatments: a margin of the original field vegetation (hereafter termed “fenced”); a rotavated and naturally regenerated margin (“rotavated”), and; a rotavated margin sown with a wildflower mixture (“seeded”). Each of these split treatments was in turn split into two management treatments (15 m each) of rotationally grazed (“grazed”) or fenced & annually mown plots (“mown”). The in‐field grazed control (“control”) plots were located adjacent to the field margin plots, in the intensively managed pasture. With the exception of the control plots, chemical inputs were excluded from all plots following establishment. Mown plots were cut annually in September to a height of 4 cm and mown vegetation was removed. The control plots were grazed in rotation with the adjacent paddock in a 21‐day rotation.

In this study, we investigated a subset of these treatments in the main experiment. Here, the emergence of invertebrates from the three field margin establishment treatments of only the mown plots (“fenced,” “rotavated” and “seeded”) were compared to the grazed control (“control”). Thus, all field margin plots sampled were mown annually, except the control plots. Width of plot was not investigated here and, as such, the emergence traps (more details see below) were randomly allocated over all widths for establishment treatments, making nine replicate plots available per establishment method. Five traps were randomly allocated to five of the nine replicates. Due to the local impact of the emergence trap, reduced light, and some damage to vegetation, the trap was moved to a new plot at the start of each sampling period within these nine replicates.

Invertebrates were sampled during six 28‐day periods over 2 years. Traps were set for 28 days and collected on the following dates: 22/05/07, 19/06/07, 17/07/07, 20/05/08, 17/06/08, and 15/07/08. In some sampling periods the emergence trap contents were lost due to a variety of reasons, including cattle interference and flooding; however, there was a minimum of three traps per treatment collected for all sampling occasions.

### 2.3 Emergence trap design

**Figure 1:**
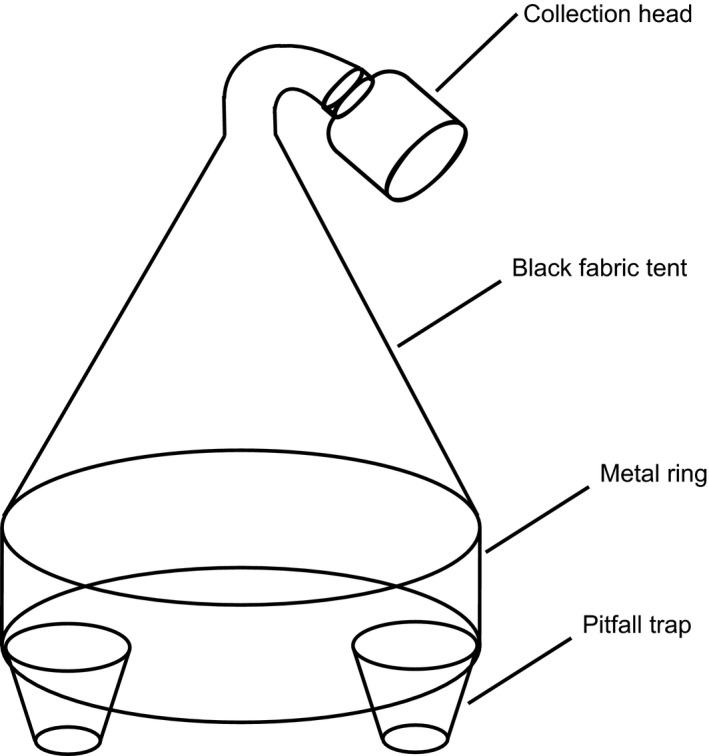
Diagram of emergence trap showing the location of pitfalls and collection head

Emergence traps are designed to catch invertebrates living in and emerging from the vegetation/soil underneath the trap. They are an effective method to catch a range of less mobile invertebrates groups, such as Diptera, Araneae, Hemiptera, and parasitic Hymenoptera. Emergence traps consisted of a metal frame with a solid metal base (diameter of 0.5 m). A black cotton tent enclosed the unit, with a collection head located at the apex of the tent. Two pitfall traps were installed on the inside edges of the emergence trap base to remove epigeic predators, such as carabid beetles, and prevent predation of newly emerged insects (Sunderland et al., 1995; Fig. 1, photograph in Fig. S2). The area sampled under the trap was 0.2 m^2^. During installation the metal base was pushed into the soil to a depth of approximately 2 cm to completely enclose the sampled area. The total monthly catch of the emergence trap, comprising the contents of the collection head and the two pitfall traps, were pooled for analysis.

### 2.4 Invertebrate identification

Invertebrate samples were sorted and specimens counted and identified to Order. Araneae were identified to species level (Roberts, 1985). Within the Hemiptera, the Auchenorrhyncha were identified to species level and, due to the large number of immature specimens, the Heteroptera were identified to family level (following Helden, Anderson, & Purvis, 2008). The parasitic Hymenoptera were identified to genus (following Anderson et al., 2008).

### 2.5 Data analysis

In this experiment, the three invertebrate groups identified to a higher level were analyzed separately (Araneae, Auchenorrhyncha and parasitic Hymenoptera). Taxon richness for each of these groups was defined as the total species (or genus) richness collected per month per trap. The sum of all individuals collected, including orders that were not identified to a higher taxonomic level (e.g., Coleoptera, Diptera, Isopoda, Dermaptera), was defined as “invertebrate abundance.” Data from traps were analyzed per trap per month.

Invertebrate abundance data was analyzed using generalized linear mixed models (GLMM), assuming a Poisson distribution, as recommended by O'Hara and Kotze (2010) for count data. Models were fitted using the GLIMMIX procedure (SAS 9.1.3, SAS Institute Inc., Cary, NC, USA). The models included the fixed effect of treatment on the abundance of Araneae, Hemiptera, parasitic Hymenoptera, Coleoptera, Diptera, Isopoda, Dermaptera and total invertebrates. Analyses focused on the response across all sampling occasions, therefore sampling period was included as a random effect in order to reflect the repeated measure structure of the data. Abundance values for the Araneae and Coleoptera were analyzed using GLIMMIX Pairwise comparisons of treatments were made using Wald Chi‐square tests.

Although GLMMs allow flexibility in the distribution of the response data, they still assume that model residuals are normally distributed. The models for Hemiptera, parasitic Hymenoptera, Diptera, Isopoda, Dermaptera and total invertebrate abundance data displayed nonnormal errors, so nonparametric ranking methods were used to test for differences in treatments with sampling occasion included as a random effect, as per Brunner and Puri (2001).

Taxon richness of Araneae and parasitic Hymenoptera were also analyzed using the same methods (repeated measures with GLIMMIX, with sampling occasion included as a random effect, with a log link function and Poisson distribution) to test for differences in treatments. Data for Auchenorrhyncha species richness did not meet the assumption of an exponential distribution, so nonparametric methods were used to test for differences in treatments, as described above.

To account for multiple testing of the relationships between taxon abundance and richness (*n *= 11) of individual groups and invertebrate richness, a Bonferroni adjustment was applied to the significance level of the tests (Sauberer et al., 2004). *p* Values were compared to a significance level of 0.0045 (0.05/11).

Multivariate analysis (using CANOCO 4.5) was used to investigate the effects of treatment on structure of invertebrate communities. Two separate partial redundancy analyses (pRDA) were performed using species abundance data for Hemiptera and parasitic Hymenoptera. Data were log‐transformed and a Monte Carlo permutation test was used to test for treatment differences (reduced model, 9,999 permutations with permutations restricted to within each sampling occasion). Partial RDA was used because plots had a homogeneous composition and showed linear species responses (Leps & Smilauer, 2003). The Araneae showed a unimodal distribution, thus partial Canonical Correspondence Analysis (pCCA) was conducted in a similar manner to the pRDA described above. To visualize the invertebrate community structure principal component analysis (PCA) was performed using abundance data, sampling occasion was included as a covariate to account for temporal variation. Treatments were overlaid on the PCA diagram as supplementary environmental variables.

## 3 RESULTS

**Figure 2:**
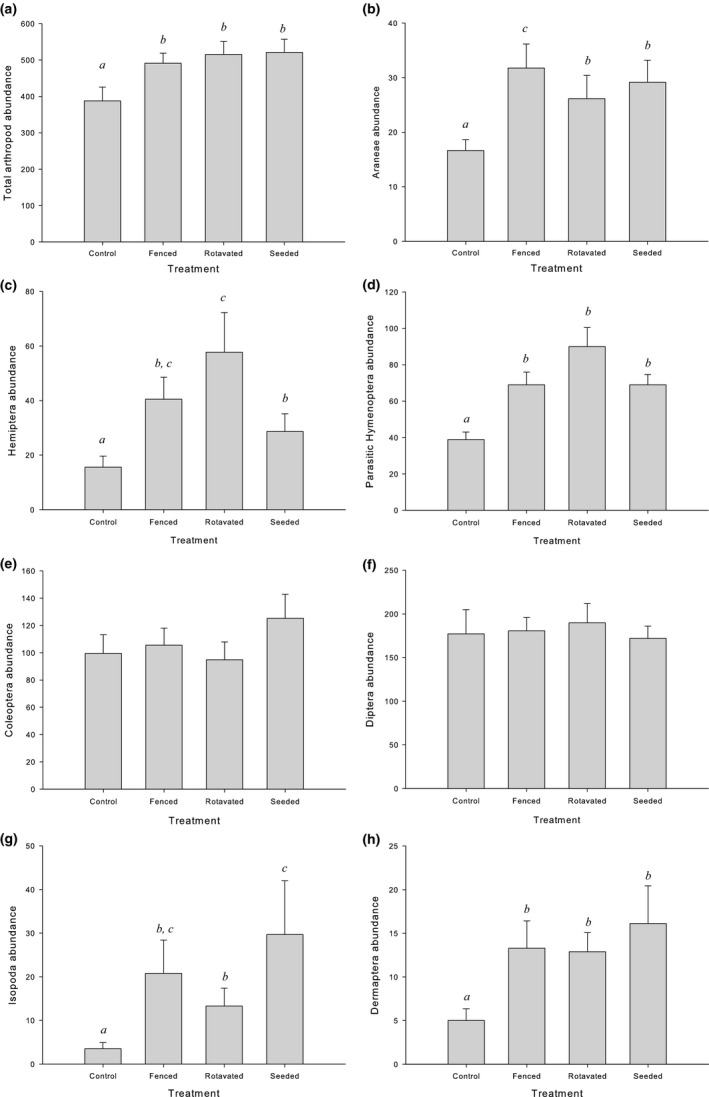
Mean abundance per emergence trap (±*SE*) of (a) all invertebrates, (b) Araneae, (c) Hemiptera, (d) parasitic Hymenoptera, (e) Coleoptera, (f) Diptera, (g) Isopoda, and (h) Dermaptera over all sampling occasions (*n* = 4–5) for different treatments. Within each taxon, treatments showing the same letter were not significantly different *(p *> .05). Note different *y*‐axis scales

A total of 42,322 invertebrate individuals were trapped over the six sampling periods. Mean abundance of each of the top seven orders recorded is provided in Fig. 2. A total of 2,902 Araneae were trapped. These included 43 species of mature spiders (*n* = 816), of which 34 were in the family Linyphiidae, four were Lycosidae, two were from the families Tetragnathidae and Theridiidae, and one species of Thomisidae. A total of 3,785 Hemiptera individuals were trapped. This included 12 species of Auchenorrhyncha and six species of Heteroptera. The Sternorrhyncha were identified to superfamily. Three superfamilies were recorded over the trapping period: Aphididae, Coccoidea, and Psyllidae. The vast majority of the Hemiptera abundance was comprised of the Aphididae (*n* = 3,327). The parasitic Hymenoptera were the most abundant and taxon‐rich group identified to a higher level. A total of 7,473 individuals were trapped comprising 132 genera from 16 families. The most taxon‐rich families recorded were *Braconidae* (33 genera), *Ichneumonidae* (25 genera), and *Pteromalidae* (20 genera). For a complete list of Araneae, Hemiptera and parasitic Hymenoptera taxa collected see Tables S1–S3, respectively.

### 3.1 Treatment effects on abundance

The individual invertebrate taxa responded differently to field margin treatments. Spider abundance was significantly higher in all treatment plots compared to the control (*p *< .0001, Table 1a, Fig. 2b). The fenced treatment contained a significantly greater abundance of spiders compared with the rotavated and seeded treatments (*p *= .006).

**Table 1:** Effects of treatment on (a) abundances of different invertebrate groups over six sampling periods calculated using GLIMMIX or nonparametric methods, (b) taxon richness of different invertebrate groups over six sampling periods calculated using GLIMMIX or nonparametric methods, and (c) different invertebrate communities using multivariate analysis with pRDA. C = Control, F = fenced, R = rotavated, S = seeded

	Pairwise comparison of treatments				Effect of treatment			
	C	F	R	S	*DF*	*F*	*p*	Sig.^a^
(a) Abundance								
Total invertebrate	*a*	*b*	*b*	*b*	(3, 106)	4.72	.0039	a
Araneae	*a*	*c*	*b*	*b*	(3, 103)	42.04	<.0001	a
Hemiptera	*a*	*b, c*	*c*	*b*	(3, 103)	10.47	<.0001	a
Hymenoptera	*a*	*b*	*b*	*b*	(3, 103)	15.87	<.0001	a
Coleoptera					(3, 103)	3.15	.028	n.s.
Diptera					(3, 101)	1.47	.214	n.s.
Isopoda	*a*	*b, c*	*b*	*c*	(3, 103)	15.94	<.0001	a
Dermaptera	*a*	*b*	*b*	*b*	(3, 103)	6.26	.0003	a
(b) Richness								
Araneae					(3, 103)	0.5	.72	n.s.
Auchenorrhyncha	*a*	*c*	*b*	*b*	(3, 103)	17.34	<.0001	a
Hymenoptera	*a*	*b*	*b*	*b*	(3, 103)	15.9	<.0001	a

	Pairwise comparison of treatments				Effect of treatment			
	C	F	R	S	Trace	*F*	*p*	Sig.
(c) Communities								
Araneae	*a*	*b*	*c*	*b*	4.7	2.27	.0001	a
Hemiptera	*a*	*b, c*	*b*	*c*	7.2	4.21	.004	a
Hymenoptera	*a*	*b*	*b*	*c*	6.7	3.35	.0001	a

a < b < c (in community data letters only indicate differences in groups).

Inf. = infinite degrees of freedom for nonparametric analysis, after Brunner and Puri (2001).

a Bonferroni adjustment for multiple comparisons, *p* < .0045 for significance.

Diptera abundance showed no response to treatment (*p *= .21). Isopod abundance was significantly affected by treatment (*p *< .0001), with seeded margins containing the greatest abundance of Isopoda (Fig. 2g). Abundance of Coleoptera was not significantly affected by field margin treatments (Fig. 2e); however, there was a trend of higher abundance in the seeded margin treatment.

Treatment was also found to significantly affect Hemipteran abundance (*p *< .0001, Table 1a, Fig. 2c) with significantly lower Hemipteran abundance recorded in the controls compared with all of the field margin treatments (*p *< .0001 for fenced, *p* = .0029 for the seeded treatment). The highest abundance of Hemiptera was recorded in the rotavated field margin treatment, and this was significantly greater than the seeded treatment (*p *= .04).

### 3.2 Treatment effects on richness

**Figure 3:**
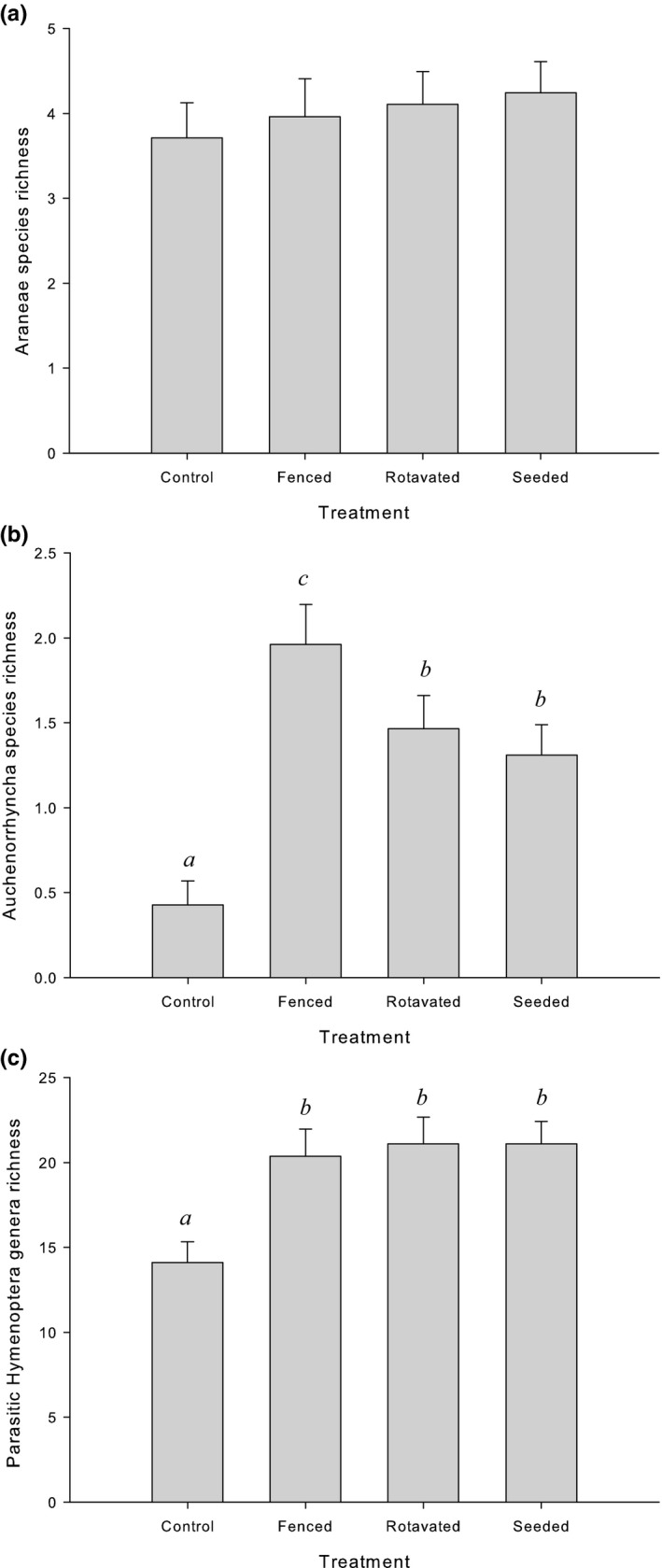
Mean taxon richness per emergence trap (±*SE*) of (a) Araneae, (b) Auchenorrhyncha, (c) parasitic Hymenoptera, over all sampling occasions (*n* = 4–5) for different treatments. Same letter denotes no significant difference in abundance (within taxa only, *p* < .05). Note different *y*‐axis scales

Richness of parasitic Hymenoptera showed a significant treatment effect (*p *< .0001, Table 1b); this was significantly greater in all field margin treatments when compared to the control (Fig. 3c, *p* < .0001 for all pairwise comparisons). However, there were no significant difference in the richness of parasitic Hymenoptera between field margin treatments.

The experimental treatment did not have a significant influence on spider species richness (*p *= .72, Fig. 3b). However, there was a significant response in Auchenorrhyncha richness (*p *< .0001, Table 1b, Fig. 3a). The fenced treatment contained the highest Auchenorrhyncha richness, and this was significantly greater than the richness in all other treatments (compared to seeded (*p *= .02), rotavated (*p *= .05), and control (*p *< .0001) respectively). Significantly higher Auchenorrhyncha richness was recorded in rotavated and seeded treatments compared with the controls (*p *< .0001 for both).

### 3.3 Treatment effects on the invertebrate communities

**Figure 4:**
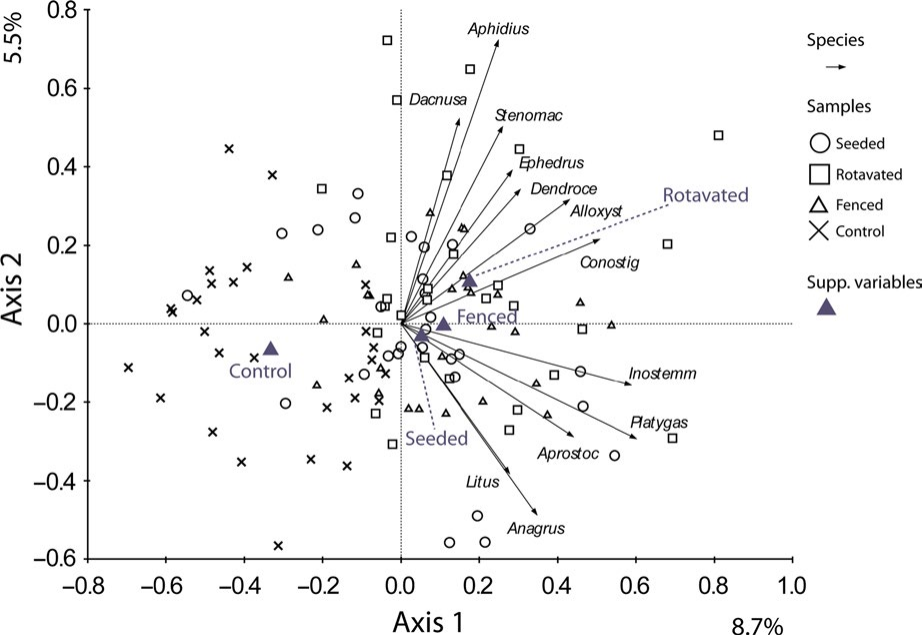
PCA ordination diagram of parasitic Hymenoptera data with samples categorized by treatment and sampling time as a co‐variable. Axis scaling is for sample scores. Eigenvalues are as percentages of variation on each axis. The 12 best‐fitting genera are shown (for abbreviations see Table S3)

For all groups, the invertebrate community of the controls were significantly different to all other field margin treatments (Table 1c). The parasitic Hymenopteran community showed three distinct groups. The community structure in fenced and rotavated treatments were similar (*p *= .23), the seeded treatment differed significantly from both of these treatment types (*p *= .0004 and .0273 respectively). These differences can be seen in the PCA ordination of the Hymenopteran community (Fig. 4), where the control plots cluster to one side of the ordination. There were no Hymenoptera genera highly associated with the control treatment, whereas there was clustering of genera associated with the other margin treatments. The genera most associated with the rotavated and fenced treatments were the braconid wasps *Aphidius, and Dacnusa,* and the Ichneumon wasp *Stenomacrus*. The Hymenoptera most associated with the seeded treatment were the *Anagrus, Litus and Aprostocetus* genera.

**Figure 5:**
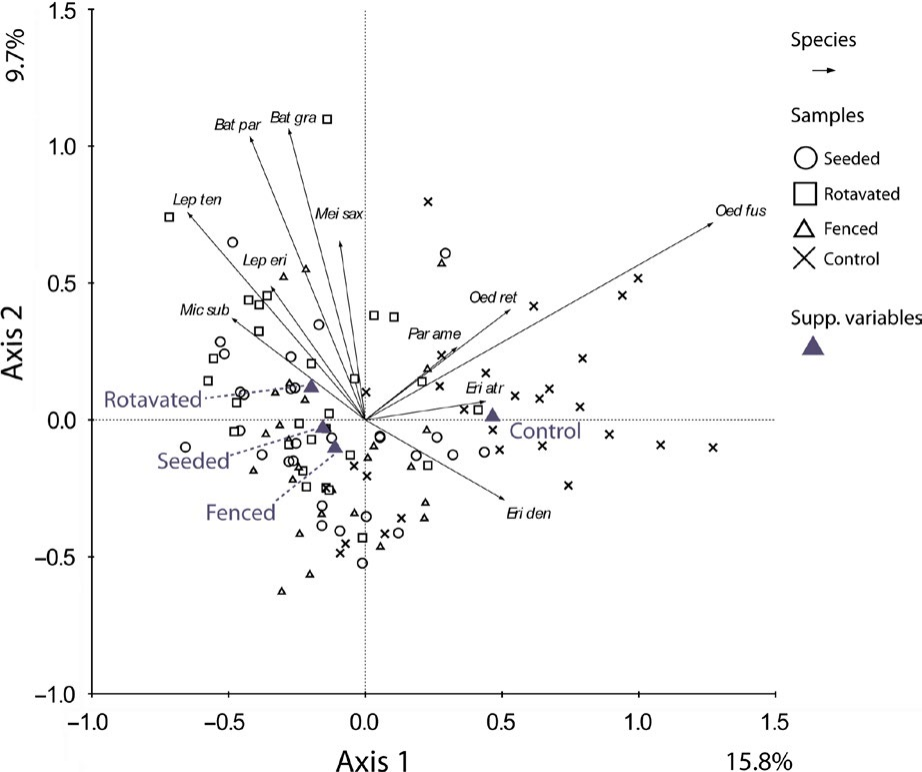
PCA ordination diagram of Araneae data with samples categorized by treatment and sampling time as a co‐variable. Axis scaling is for sample scores. Eigenvalues are as percentages of variation on each axis. The 15 best‐fitting species are shown. (For species' abbreviations see TableS1)

Significant differences between treatments were found in the spider community (Table 1c, PCA ordination Fig. 5). The controls are clearly clustering in the ordination diagram, with the field margin treatments in a separate cluster. There were no significant difference between spider communities in the fenced and seeded treatments (*p *= .6139). The spider communities from the rotavated and seeded field margins showed a marginal difference in significance (*p *= .061). In the ordination, the species which are most associated with the controls include *Oedothorax fuscus, Oedothorax retusus and Erigone dentipalpis*. The species most associated with the field margin treatments are *Tenuiphantes tenuis, Micrargus subaequalis, and Lepthyphantes ericaeus*.

**Figure 6:**
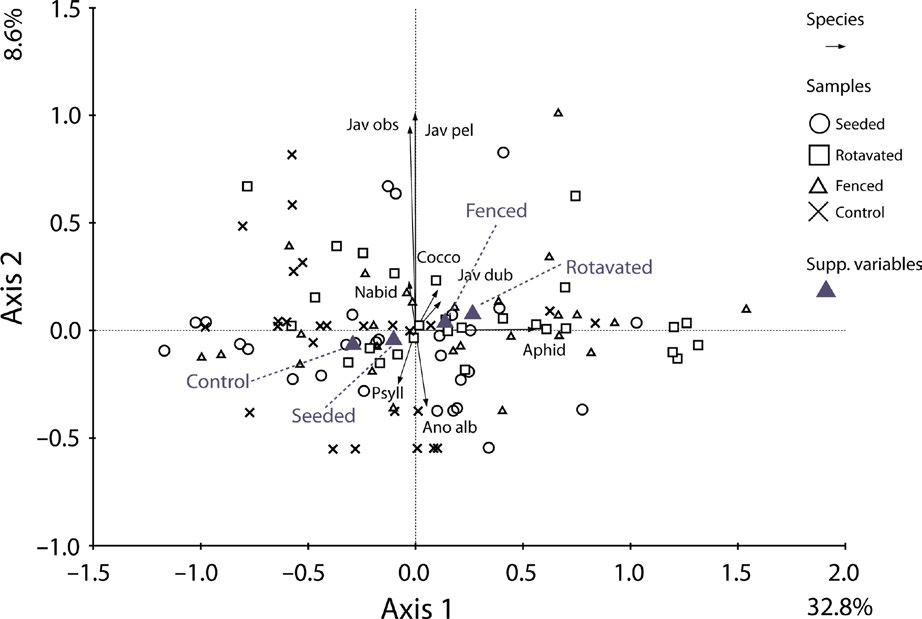
PCA ordination diagram of the Hemipteran community data with samples categorized by treatment and sampling time as a co‐variable. Axis scaling is for sample scores. Eigenvalues are as percentages of variation on each axis. The eight best‐fitting taxa are shown (for taxon abbreviations, see TableS2)

Field margin treatment affected hemipteran community structure (*p *= .0004, Table 1c, PCA ordination Fig. 6). The rotavated and seeded treatments showed significant differences in their hemipteran community structure (*p *= .013). However, the hemipteran community of the fenced treatment did not differ from that of the rotavated (*p *= .43) or seeded (*p *= .071) treatments (Table 1c).

## 4 DISCUSSION

In comparison to the grazed controls, field margin establishment treatment (regardless of treatment type) resulted in an increased abundance and taxon richness of most of the invertebrate groups. These results concur with many arable field margin studies which show that providing an uncropped field margin enhances invertebrate abundance when compared to a cropped margin (Aviron, Jeanneret, Schupbach, & Herzog, 2007; Thomas & Marshall, 1999). In arable systems, leaving an uncropped field margin results in this area remaining uncut, unploughed and unsprayed with pesticide (apart from drift). However, as grasslands are generally grazed by livestock, the development of an “uncropped” margin requires the installation of fencing in order to exclude grazing, with labor and cost implications.

Exclusion of grazing (by fencing, and with annual mowing) improved both abundance and taxon richness of most of the invertebrate groups in this study. This could be due to a range of factors, including: increased sward biomass allowing for more food resources; more complex sward structure facilitating the development of a greater number of habitat niches; a reduction in disturbance from trampling and poaching, and; a change in the sward botanical composition (Morris, 2000). Grazing within this experiment was intensive, comprising a 21‐day dairy rotation that was associated with little development of sward structure, high levels of disturbance, limited amounts of standing sward biomass and a reduction in plant species richness (Fritch et al., 2011).

Seeding with a wildflower mixture was the most successful establishment method to enhance plant species richness and this effect persisted throughout the 7 years of the experiment (*x* = 16.4 ± 0.43 *SE* plant species richness per 1 × 3 m^2^ quadrat, Fritch et al., 2011). Fenced (*x* = 6.01 ± 0.30 *SE*) and rotavated (*x* = 9.7 ± 0.34 *SE*) treatments contained significantly fewer plant species; grazed controls contained 9.83 ± 0.24 species (Fritch et al., 2011).

However, the invertebrate groups analyzed responded differently to different field margin treatments. For example, Isopod numbers were highest in fenced and seeded margins, whereas spider abundance was greatest in fenced margins. Abundance of coleoptera was not affected by field margin treatment. These results concur with other studies of beetles in field margin habitat (Smith, Potts, Woodcock, & Eggleton, 2008; Woodcock, Westbury, Potts, Harris, & Brown, 2005). However, both Thomas and Marshall (1999) and Asteraki, Hart, Ings, and Manley (2004) found increased beetle abundance in sown field margins compared with those with grass species.

Several studies have found a positive relationship between invertebrate abundance and plant species richness, with invertebrate abundances being higher in sown plots, compared to low‐diversity naturally regenerated plots. However, many of these studies focused on transient species feeding in field margins, e.g., bumblebees and butterflies (Meek et al., 2002; Potts et al., 2009; Pywell et al., 2006). By using emergence traps to focus on resident invertebrate taxa emerging from vegetation and soil, transient species breeding in habitats other than the field margins were not included. The major benefit in this study is that the changes in abundance and diversity of invertebrates can be attributed to the modified management of the field margins.

Management practices aimed at enhancing invertebrate taxon richness and abundance may have to be balanced against those aimed at improving botanical diversity in field margins. An understanding of the relationships between plant diversity and invertebrate diversity is necessary for the successful design and implementation of agri‐environment measures. Conservation aims should be clearly defined before specific management regimes are chosen and implemented. In the experimental design and management regime of this experiment, management for plant diversity was given priority. Regular mowing or grazing of grassland is often required for conservation of plant diversity (Vickery, Feber, & Fuller, 2009), but uncut or low‐intensity grazing of vegetation is beneficial for many invertebrates. Likewise, leaving the mown material in situ provides a habitat for invertebrate populations (Baines, Hambler, Johnson, Macdonald, & Smith, 1998). Non‐removal of mown vegetation may benefit invertebrates; however, over a longer time‐frame, it tends to increase soil nutrient status and competitive plant species become dominant resulting in a less species‐rich sward (Vickery et al., 2009). In this experiment, plots were mown annually and all vegetation was removed. The aim of this management was to maintain plant species richness. However, this management may have resulted in the removal or mortality of invertebrates at their various life stages, such as aestivating eggs, pupae, and larvae, and the removal of their microhabitats. For example, spider species richness did not differ significantly between the controls and treatments. All treatments had low amounts of aboveground biomass over winter, thus providing little potential in terms of overwintering habitat. This was due to annual mowing of the vegetation in September to a height of 4 cm and removal of the harvested biomass. Schmidt, Rocker, Hanafi, and Gigon (2008) found that the abundance of specific spider families was enhanced in rotationally cut fallow areas within mown grasslands, when compared to annually mown grassland. Carabid diversity was also enhanced in uncut grassland margins, compared with grassland which was cut one to three times a year (Haysom et al., 2004).

One drawback (and advantage) to emergence trapping is that many of the trapped individuals are immature and cannot be identified to a lower taxonomic level. While the low number of mature individuals identified to species level hinders analysis based on species richness, high numbers of immature individuals favors the analysis of taxonomic group abundance. Spiders were a good example of this, because the majority of individuals trapped were immature. Therefore, abundance, as opposed to richness, of a group (including immature individuals) may be a good measure of the effectiveness of a treatment. Spider abundance was greatest in the plots that had been fenced only, i.e., not rotavated. However, species richness did not vary between field margin treatments or controls. These results contradict those of Baines et al. (1998), who found that sown margins gave rise to greater abundance and richness of spiders compared to natural regeneration.

Hemipteran abundance, which was mostly attributable to aphids, was greatest in the fenced and rotavated margins; however, species richness (of only the Auchenorrhyncha and Heteroptera) was highest in the fenced margins. This concurs with the finding of Petermann, Müller, Weigelt, Weisser, and Schmid (2010) who concluded that aphid abundance is driven by sward biomass and that the highest abundances are found at intermediate plant species richness. Specialist herbivores, such as aphids, are predominantly controlled by the density and richness of their host plants (Joshi et al., 2004).

Hymenopteran parasitoids may be reliable indicators of arthropod diversity, as they are predators and parasitoids of a range of other arthropod groups (Quicke, 1997). Anderson et al. (2011) found that parasitic Hymenoptera were reliable indicators of invertebrate diversity in intensively managed grassland swards. However, in this study, both the richness and abundance of parasitic Hymenoptera was similar across all field margin treatments when grazing was excluded. Ó hUallacháin et al. (2014) examined parasitic Hymenoptera on these experimental plots using suction sampling over a 5 year period. The findings of this present study concur with Ó hUallacháin et al. (2014) and suggest that the total invertebrate species richness did not vary between treatments; however, the community structure was distinctive, and dependent on the treatment.

The habitat heterogeneity theory predicts that a diverse environment provides more niches, thereby increasing species diversity (Tews et al., 2004). In this experiment, no single field margin treatment could be identified as the best in terms of its ability to support overall invertebrate richness. However, one must look further than simple species richness and examine the associated invertebrate communities. For example, the parasitic Hymenopteran community formed three distinct groups: the control, the fenced/rotavated, and the seeded community. The genera dominating these communities parasitize different groups of invertebrates. For example, the genera most associated with the rotavated and fenced treatments were the braconid wasps, *Aphidius*, and *Dacnusa*, and the Ichneumon wasp *Stenomacrus*. These genera are parasitoids of aphids, plant mining Diptera larvae, and fungus gnat respectively (Ó hUallacháin et al., 2014). The Hymenoptera most associated with the seeded treatment were the *Anagrus*,* Litus* and *Aprostocetus* genera. These are chalcid wasps, which parasitize arthropod eggs (Ó hUallacháin et al., 2014) and are used in biological control of agricultural pests. To conserve the full complement of species recorded throughout the duration of the experiment, and their functions, all sward types are required. This maximizes the beta diversity between habitats and allows the conservation of most species. Where one farm contains all three sward types the overall beta diversity of parasitic Hymenoptera is expected to be higher.

A possible limitation of this experiment was the low colonization potential from a potentially restricted invertebrate community in the surrounding landscape. In intensive agricultural landscapes with little or no semi‐natural habitat, the invertebrate communities of newly created habitats (such as field margins) are limited by the available species pool in the landscape and their dispersal abilities (Steffan‐Dewenter & Tscharntke, 2002). The study site was located in an intensively managed agricultural landscape, with little semi‐natural habitat in proximity to the study area, e.g., no hedgerows are present and fields are separated by electric wire. In addition, colonization could be counteracted by the annual mowing of the plots, (that removed overwintering habitats and microhabitats for invertebrates), requiring field margins to be re‐colonized annually from other habitats present in the surrounding landscape. Differences between field margin treatments might be limited by the available pool of potential colonizers of the margin plots; nevertheless, we found significant differences between treatments, mainly due to the removal of grazing.

This research is relevant to the design of practical agri‐environment measures for intensively managed grasslands. We demonstrate the capacity of field margin establishment to increase the abundance and richness in nearly all sampled invertebrate groups on previously more depauperate areas of intensively managed grassland. These results from grassland field margins provide evidence to support practical actions that can inform future options for Greening (Pillar 1) and agri‐environment measures (Pillar 2) of the CAP. This comes at a time of intense discussion regarding CAP reform, and evidence such as this is needed to inform policy. Although we found that managed grassland field margins enhanced the diversity and abundance and changed the community structure of multiple invertebrate groups that we sampled, we did not investigate the influence of these communities on ecosystem function. If agri‐environment measures for grassland field margins aim to support higher ecosystem function in farmland, greater clarity is needed on: the level of invertebrate density required for provision of ecosystem services, and; the density of pasture field margins/semi‐natural habitats needed for field margins to help attain the desired levels of invertebrate diversity (e.g., Dainese, Luna, Sitzia, & Marini, 2015).

Overall, an exclusion of grazing, by fencing, improved both the abundance and species richness of most of the groups in this study. No single field margin treatment was best for overall invertebrate abundance and richness, as each sampled taxonomic grouping responded differently. Each field margin treatment supported a distinct resident invertebrate community. Thus, the use of a range of methods to establish ungrazed field margin habitats should support the highest resident invertebrate diversity on grassland farms. Management practices for invertebrate conservation often contradict those for botanical conservation (Konvicka et al., 2008). Conservation aims of agri‐environment measures should be clearly defined before specific management regimes are chosen and implemented.

## AUTHOR CONTRIBUTION

HS and JF designed the field experiment. JF, HS, RF, and DOhU contributed to the conception and/or design of this study. RF designed the sampling method; RF, DohU, and SM collected the data; RF analyzed the data and drafted the manuscript; all authors critically revised the manuscript and approved of the version to be published.

## CONFLICT OF INTEREST

None declared.

## DATA ACCESSIBILITY

Data will be archived in Dryad Digital Repository (doi:10.5061/dryad.92b1f) and TStór, an Open Access repositorymaintained by Teagasc, the Agriculture and Food Development Authority in Ireland (http://t-stor.teagasc.ie/).
