# Supplementary material for: Enhancing the diversity of breeding invertebrates within field margins of intensively managed grassland: Effects of alternative management practices
Source: Ecol Evol. 2017 Oct 19;7(22):9763–74. doi: 10.1002/ece3.3302 (PMC5696416; doi:10.1002/ece3.3302)
Supplement: Supplementary file 1 [file ECE3-7-9763-s001.docx]

Supplementary Materials


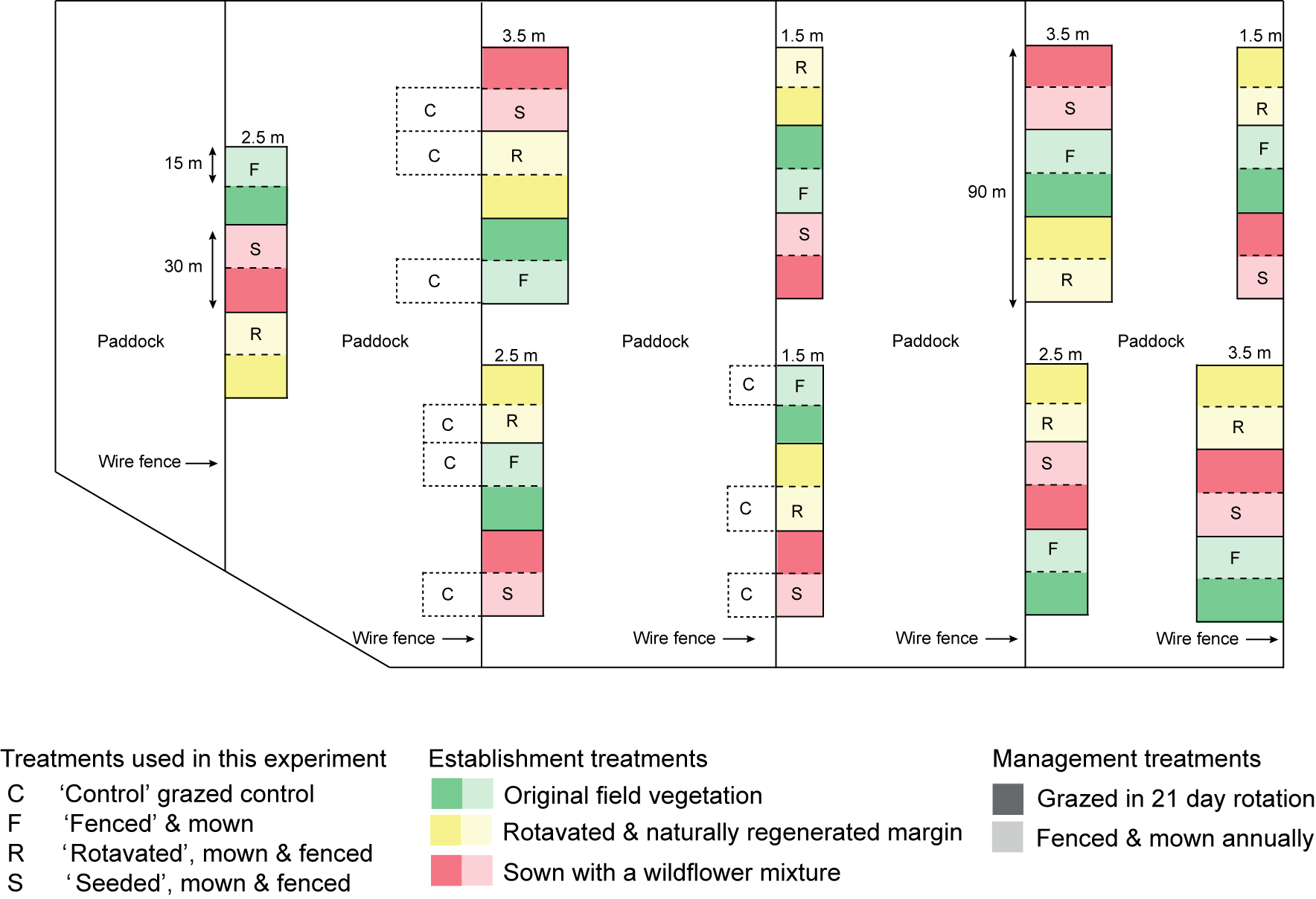


Fig. S1. Diagram of experimental design and plot locations (not to scale), darker shading indicates plots grazed on a 21 day rotation, lighter shading indicates plots which are fenced and mown annually. This diagram presents the full experimental design of a larger field experiment, as reported in Fritch et al. (2011). The current study used a subset of these treatments and plots, as indicated by C, F, R, and S for control, fenced, rotavated and seeded treatments, respectively.

Fritch, R.A., Sheridan, H., Finn, J.A., Kirwan, L. & Huallachain, D.O. (2011) Methods of enhancing botanical diversity within field margins of intensively managed grassland: a 7-year field experiment. *Journal of Applied Ecology,* **48,** 551-560.


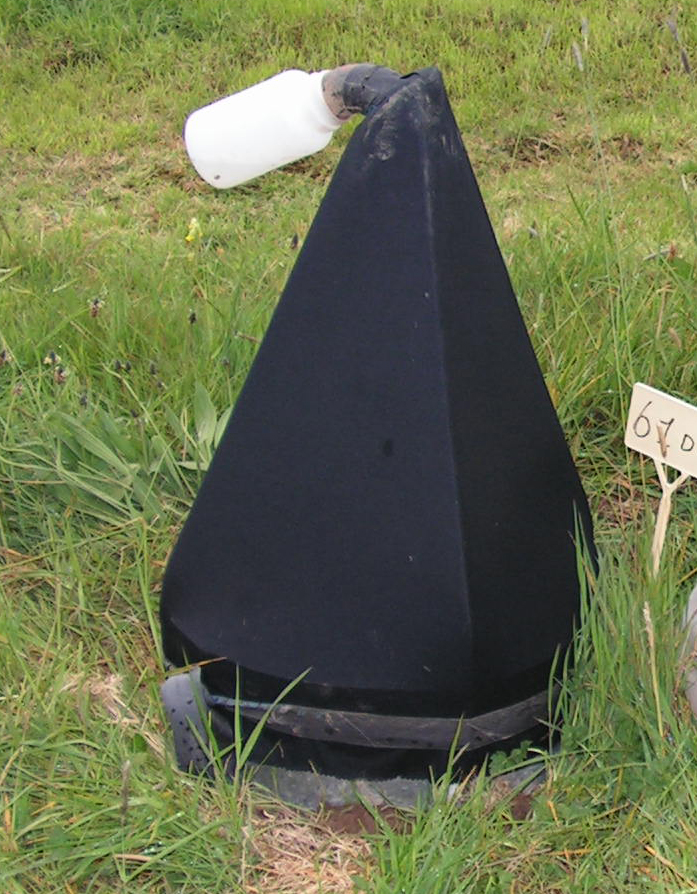


Fig. S2. Photograph of an emergence trap installed in the field site.

Table S1. Spider species collected from all sampling occasions, abbreviations for ordinations in brackets.

| *Agyneta decora* |
| --- |
| *Agyneta rurestris* |
| *Agyneta saxatilis* (Agy sax) |
| *Alopecosa pulverulenta* |
| *Bathyphantes approximatus* |
| *Bathyphantes gracilis* (Bat gra) |
| *Bathyphantes parvulus* (Bat par) |
| *Centromerita bicolor* |
| *Dicymbium nigrum* |
| *Dicymbium tibiale* |
| *Enoplognatha ovata* |
| *Erigone atra* (Eri atr) |
| *Erigone dentipalpis* (Eri den) |
| *Erigonella hiemalis* |
| *Gongylidiellum vivum* |
| *Hypomma bituberculatum* |
| *Leptorhoptrum robustum* |
| *Micrargus subaequalis* (Mic sub) |
| *Monocephalus fuscipes* |
| *Oedothorax agrestis* |
| *Oedothorax fuscus* (Oed fus) |
| *Oedothorax gibbosus* |
| *Oedothorax retusus* (Oed ret) |
| *Ostearius melanopygius* |
| *Pachygnatha clercki* |
| *Pachygnatha degeeri* |
| *Palliduphantes ericaeus* |
| *Pardosa amentata* (Par ame) |
| *Pardosa palustris* |
| *Pardosa pullata* |
| *Pocadicnemis juncea* |
| *Pocadicnemis pumila* |
| *Porrhomma errans* |
| *Porrhomma montanum* |
| *Porrhomma pygmaeum* |
| *Robertus lividus* |
| *Savignia frontata* |
| *Tenuiphantes tenebricola* (Ten eri) |
| *Tenuiphantes tenuis*(Ten ten) |
| *Tenuiphantes zimmermanni* |
| *Walckenaeria acuminata* |
| *Walckenaeria vigilax* |
| *Xysticus cristatus* |

Table S2. Hemiptera species/pseudo-species collected from all sampling occasions, abbreviations for ordinations in brackets.

| **Identification level** | **Species or pseudo-species** |
| --- | --- |
| Family | Anthocoridae |
| Family | Lygaeidae |
| Family | Miridae |
| Family | Nabidae |
| Family | Saldidae |
| Superfamily | Aphididae (Aphid) |
| Superfamily | Coccoidea (Cocco) |
| Superfamily | Psyllidae (Psyll) |
| Species | *Neophilaenus lineatus* |
| Species | *Philaenus spumarius* |
| Species | *Anoscopus albifrons/duffieldi* (Ano alb) |
| Species | *Macrosteles* sp |
| Species | *Megophthalmus scanicus* |
| Species | *Criomorphus albomarginatus* |
| Species | *Dicranotropis hamata* |
| Species | *Javesella dubia* (Jav dub) |
| Species | *Javesella obscurella* (Jav obs) |
| Species | *Javesella pellucida* (Jav pel) |
| Species | *Muellerianella* sp |
| Species | *Paraliburnia clypealis* |

Table S3. Parasitoid Hymenoptera genera collected from all sampling occasions, abbreviations for ordinations in brackets.

| *Aclastus* |  | *Demopheles* |
| --- | --- | --- |
| *Aclista* |  | *Dendrocerus* (Dendroce) |
| *Alloea* |  | *Diglypus* |
| *Alloxysta* (Alloxyst) |  | *Dinotrema* |
| *Alysia* |  | *Ephedrus* (Ephedrur) |
| *Amphibulus* |  | *Eusterinx* |
| *Anagrus* (Anagrus) |  | *Ganaspis* |
| *Anaphes* |  | *Gastrancistrus* |
| *Anteon* |  | *Gelis* |
| *Apanteles* |  | *Gonatocerus* |
| *Aphanogmus* |  | *Halticoptera* |
| *Aphelinus* |  | *Hemiptarsenus* |
| *Aphidius* (Aphidius) |  | *Ichneumon* |
| *Aprostocetus* (Aprostoc) |  | *Ichneumonidae* spp *A** |
| *Aritranis* |  | *Ichneumoninae* spp *B** |
| *Asaphes* |  | *Ichneumoninae* spp *C** |
| *Asecodes* |  | *Ichneumoninae* spp *E** |
| *Aspilota* |  | *Idiotypa* |
| *Baeus* |  | *Inostemma* (Inostemm) |
| *Basalys* |  | *Ischnus* |
| *Belyta* |  | *Janssoniella* |
| *Binodoxys* |  | *Kleidotoma* |
| *Blacus* |  | *Lagynodes* |
| *Bracon* |  | *Lamennaisia* |
| *Callitula* |  | *Leptacis* |
| *Centistes* |  | *Leptomastix* |
| *Ceraphon* |  | *Leptopilina* |
| *Chasmodon* |  | *Litus* (Litus) |
| *Chlorocytus* |  | *Macrocentrus* |
| *Chorebus* |  | *Macroglenes* |
| *Codrus* |  | *Megastylus* |
| *Coelinius* |  | *Meraporus* |
| *Conostigmus* (Conostig) |  | *Mesoleptus* |
| *Cothonaspis* |  | *Mesopelobus* |
| *Cremnodes* |  | *Meteorus* |
| *Cryptinae B* |  | *Microplitis* |
| *Cryptinae C* |  | *Miota* |
| *Cryptopimpla* |  | *Miscogaster* |
| *Cubocephalus* |  | *Monelata* |
| *Cyrtogaster* |  | *Monoctonus* |
| *Dacnusa* (Dacnusa) |  | *Omphale* |
| *Dapsilarthra* |  | *Ooctonus* |

*Ichneumonidae were identified to pseudo-species

| *Opazon* |  | *Rhoptromeris* |
| --- | --- | --- |
| *Opius* |  | *Rhopus* |
| *Orthizema* |  | *Rhorus* |
| *Orthocentrus* |  | *Seladerma* |
| *Orthostigma* |  | *Semiotellus* |
| *Panstenon* |  | *Spalangia* |
| *Paramesius* |  | *Spaniopus* |
| *Pediobius* |  | *Spilomicrus* |
| *Pentapleura* |  | *Stenomacrus* (Stenomac) |
| *Perilitis* |  | *Stenomalina* |
| *Peristenus* |  | *Sympiesis* |
| *Phaenocarpa* |  | *Synacra* |
| *Phaenoglypis* |  | *Synopeas* |
| *Phobocampe* |  | *Syrphoctonus* |
| *Phygadeuon* |  | *Telenomus* |
| *Platygaster* (Platygas) |  | *Tetramesa* |
| *Pleolophus* |  | *Theroscopus* |
| *Pnigalio* |  | *Thrybius* |
| *Polynema* |  | *Toxeuma* |
| *Praon* |  | *Trybliographa* |
| *Promethes* |  | *Trichomalopsis* |
| *Psichacra* |  | *Trichopria* |
| *Psyllaephagus* |  | *Trimoris* |
| *Pteromalus* |  | *Tymmophorus* |
